# *Rhodococcus equi* and *Brucella* pulmonary mass in immunocompetent: A case report and literature review

**DOI:** 10.1515/biol-2022-0888

**Published:** 2024-06-18

**Authors:** Pengfei Li, Lifang Zhang, Xicheng Li, Xuejuan Zhang

**Affiliations:** Department of General Medicine, The Affiliated Hospital of Qingdao University, Qingdao, Shandong, China

**Keywords:** *Rhodococcus equi*, *Brucella*, pulmonary occupancy, *Brucella*r spondylitis

## Abstract

*Rhodococcus equi*, predominantly recognized as an opportunistic pathogen affecting immunocompromised hosts, and *Brucella*, a widespread zoonotic bacterium, infrequently co-infect immunocompetent adults, thereby posing a distinctive diagnostic challenge. Here, we describe a case involving a 53-year-old male with a history of goat farming, who presented with persistent chest tightness, cough, and notable weight loss, absent fever. Radiological and bronchoscopic assessments showed a right hilar mass, extensive vertebral destruction, and bronchial lesions, deviating from the typical symptoms associated with either pathogen. Laboratory analyses confirmed a co-infection involving *R. equi* and *Brucella*. Initial therapy with levofloxacin and vancomycin proved ineffective; however, a subsequent treatment regimen comprising azithromycin, etimicin, minocycline, and moxifloxacin resulted in substantial clinical improvement. This case accentuates the intricacies involved in diagnosing and managing atypical co-infections in immunocompetent individuals and underscores the importance of careful microbiological testing to inform effective therapeutic strategies.

## 1 Introduction

*Rhodococcus equi*, an infrequently encountered opportunistic pathogen, predominantly afflicts neonatal foals, resulting in chronic pyogenic bronchopneumonia [1]. This infection predominantly manifests in immunocompromised hosts, such as individuals with HIV, characterized by pulmonary consolidation and multiple cavitations, yet remains unusual in immunocompetent persons [2]. Conversely, Brucellosis, a zoonotic disease caused by the *Brucella* species, primarily *Brucella melitensis* and *Brucella abortus*, manifests in a diverse array of clinical symptoms, including fever, night sweats, myalgia, arthralgia, and general malaise [3]. In this case study, an immunocompetent individual distinctively presented with coexisting infections of *R. equi* and *Brucella*. The patient demonstrated radiological evidence of an isolated pulmonary mass at the hilum, extensive vertebral destruction, and bronchial lesions evident on bronchoscopy. Notably, the patient lacked the cardinal symptom of brucellosis: fever. These multifocal presentations highlight the diagnostic challenge and the need for comprehensive investigation in atypical infectious cases.

## 2 Case presentation

A 53-year-old male presented at the Affiliated Hospital of Qingdao University reporting a 2-month history of persistent chest tightness and cough, sporadically accompanied by chest and significant back pain. Notably, he denied any fever or hemoptysis. Since the initial manifestation of symptoms, the patient has retained full consciousness and demonstrated stable mental acuity, alongside a normal appetite, adequate sleep quality, and consistent bowel and urinary functions; importantly, he has experienced a significant weight reduction of approximately 10 kg over the past month. Prior to this illness, the patient reported a history of generally good health, with brief engagement in goat farming and an incident where he was bitten by a goat.

### 2.1 Physical examination

During the examination, the patient remained alert and mentally stable. Pronounced coarse breath sounds were observed in both the upper and lower regions of the right lung. Audible dry and moist rales were present, with a predominance of moist rales in the lower lobes. The cardiac rhythm was regular, and no abnormal murmurs were detected upon auscultation of any heart valve areas, indicating normal cardiac function. The abdomen was soft and non-tender with no rebound tenderness. No masses were palpable, and neither the liver nor spleen was felt below the ribs, suggesting an absence of significant abdominal pathology. Additionally, bowel sounds were audible at a normal rate of four per minute, with no shifting dullness, indicative of normal gastrointestinal activity. Finally, no edema was observed in the lower extremities, and muscle tone in all limbs was normal, suggesting no significant neuromuscular or vascular complications.


**Informed consent:** Informed consent has been obtained from all individuals included in this study.
**Ethical approval:** The research related to human use has been complied with all the relevant national regulations, institutional policies, and in accordance with the tenets of the Helsinki Declaration and has been approved by the Ethics Committee of the Affiliated Hospital of Qingdao University.

### 2.2 Laboratory test

Blood routine results: Hemoglobin 101 g/L (130–175 g/L); C-reactive Protein: 19.61 mg/L (0–5 mg/L); Procalcitonin: 0.072 ng/mL (<0.05 ng/mL); CD4 Absolute Count: 427.00 cells/µL (544–1,212 cells/µL); Creatinine 219 µmol/L (79–133 µmol/L); Sputum Culture and Identification: Candida glabrata; *Brucella* Coombs Test: *Brucella* IgG Antibody: Positive, Brucellosis Tests: Tube Agglutination Test: 1:200 (++++), Tiger Red Plate Method: Positive; Tests for ANCA, ENA Antibody Spectrum, ANA, and Tumor Marker Screening revealed no significant abnormalities.

### 2.3 Imaging examination

**Figure 1:**
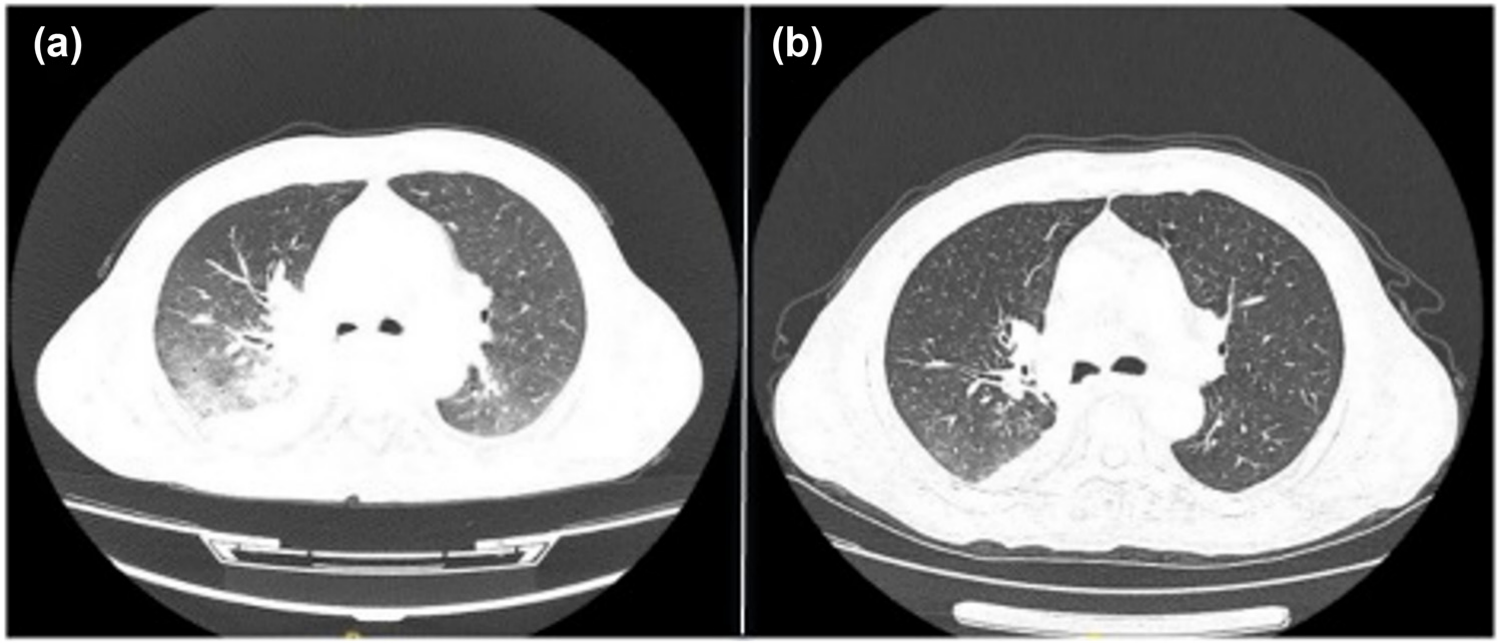
(a) CT scan showing a right hilar mass with features indicative of obstructive pneumonia. (b) Follow-up CT scan exhibits decreased size of the right hilar mass and improved lung consolidation, indicative of treatment response.

**Figure 2:**
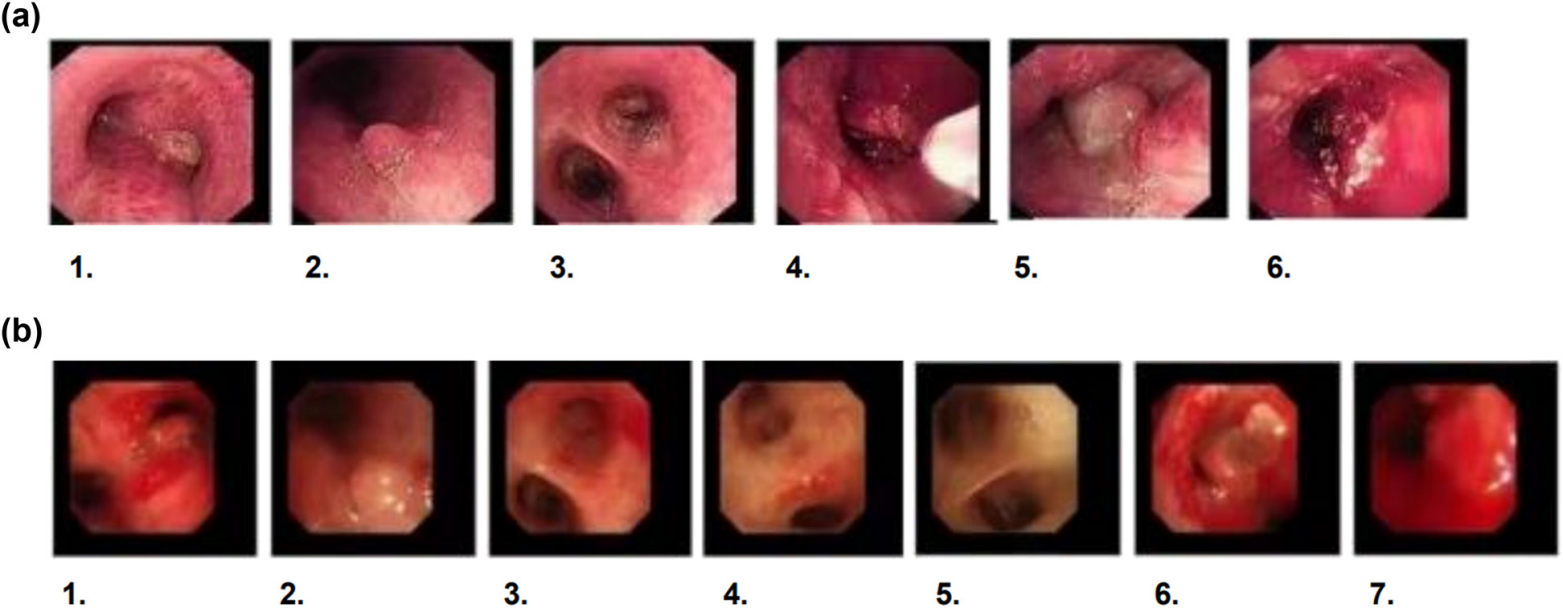
(a) The initial bronchoscopy reveals lesions in both the left and right bronchi and that electrocautery was performed. 1. Tracheal carina, 2. Left Main Bronchus, 3. Distal Left Main Bronchus, 4. Electrocautery in Progress, 5. Distal Right Main Bronchus, 6. Right Main Bronchus Opening Under Treatment. (b) The follow-up bronchoscopy shows that despite the intervention, there are still lesions present. 1. Tracheal carina, 2. Left Main Bronchus, 3. Distal Left Main Bronchus, 4. Left Upper Lobe Bronchus Opening, 5. Left Lower Lobe Bronchus Opening, 6. Right Main Bronchus Opening, 7. Right Main Bronchus Opening Under Treatment.

**Figure 3:**
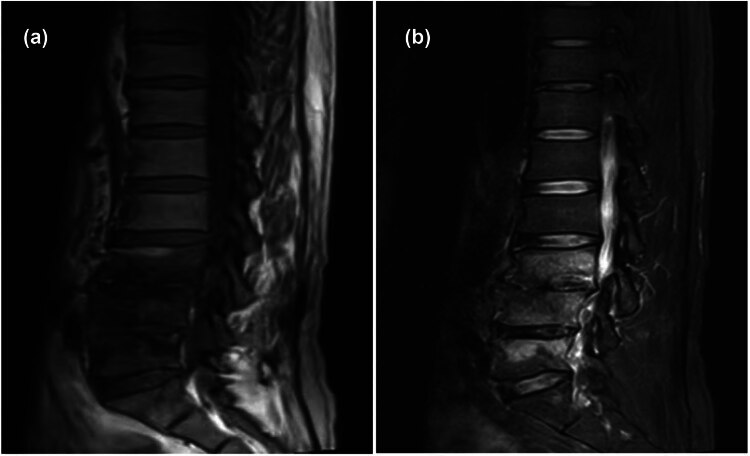
Lumbar spine MRI indicates abnormal signal intensity in the L3–L5 vertebral bodies with low signal on T1-weighted images (a) and high signal on T2-weighted images (b), suggestive of an infectious lesion.

Initial high-resolution computed tomography (CT) scan of the chest at admission revealed right hilar occupation, extensive mediastinal lymphadenopathy, minimal bilateral pleural effusions, coronary artery calcification, and slight pericardial effusion, along with a high-density shadow localized to approximately the T7 vertebra (Figure 1a). Pre-discharge CT re-evaluation demonstrated marked improvement relative to prior findings (Figure 1b). During the initial bronchoscopy, examination of the lower tracheal segment revealed notable findings including fish-scale-like alterations on the mucosa, a blunted carina, and infiltrative changes, suggesting extensive mucosal involvement. An abnormal growth completely obstructed the lumen at the opening of the right main bronchus, while another lesion was identified on the wall of the left main bronchus, with its distal end remaining patent. Electric loop excision was performed on the right main bronchus to remove the obstructing lesion. This procedure revealed extensive abnormal extension to the distal end of the bronchus, obscuring the openings of the upper and lower lobes. Subsequent irrigation of the bronchus was performed to ensure cleanliness and assess clearance (Figure 2a). A follow-up bronchoscopy conducted a week later revealed polypoid hyperplasia on the inner wall of the left main bronchus and a spherical lesion at the opening of the right main bronchus. Following snare excision of the right bronchial lesion, further examination observed that the growth extended to the terminal end of the bronchus (Figure 2b). Lumbar spine MRI findings: Abnormal signal in L3–L5 vertebrae, suggesting infectious lesions, with posterior spondylolisthesis of L3 and L4 vertebrae (Grade I) and degenerative changes in the lumbar spine (Figure 3).

### 2.4 Diagnosis

Bronchoscopic biopsy of the right main bronchus revealed extensive inflammatory granulation tissue containing a mixture of histiocytes, lymphocytes, plasma cells, and neutrophils. This suggests a robust inflammatory response. Immunohistochemical analysis and special staining, including positive Silver and PAS reactions, supported the presence of an infection, strongly suggestive of *R. equi*, corroborated by spore observation. A CT-guided lung biopsy confirmed chronic inflammation along with alveolar epithelial hyperplasia. Next-generation sequencing of bronchoalveolar lavage fluid definitively identified *R. equi* with high read counts. Concurrent serological testing yielded positive results for *Brucella*, consistent with the patient’s history and clinical manifestations.

### 2.5 Treatment and prognosis

Initially, the patient was treated with a regimen comprising levofloxacin and vancomycin, which yielded a negligible therapeutic response. Subsequently, treatment was transitioned to a combination of azithromycin and etimicin. The final therapeutic strategy comprised minocycline and moxifloxacin, augmented by adjunct treatments including antitussives, mucolytics, and bronchodilators for symptomatic relief. Budesonide inhalation therapy was utilized to suppress airway granulomatous proliferation. Concurrently, the patient received calcitriol and cholecalciferol for calcium supplementation. Upon discharge, the patient demonstrated significant amelioration of cough symptoms and substantial improvement in chest CT imaging (Figure 1b).

## 3 Discussion

*R. equi*, classified as an aerobic actinomycete within the Nocardiaceae family, is a pleomorphic Gram-positive bacterium that exhibits partial acid-fast staining and is encountered in soil, human feces, and the feces of herbivorous animals. *R. equi* acts as a pathogen primarily affecting animals like horses and cows, typically inducing purulent bronchopneumonia in foals aged 1–6 months, while in humans, it is recognized as an opportunistic pathogen predominantly affecting individuals with compromised immune systems, with the incidence of infections escalating particularly in the context of widespread HIV/AIDS [4,5,6]. *R. equi* infections are primarily contracted through inhalation, leading to pulmonary afflictions, or via wounds, resulting in subcutaneous infections and potentially bacteremia or sepsis. Although primarily responsible for pulmonary infections, *R. equi* occasionally manifests in other organs including the liver, brain, and small intestine [7,8,9]. Its pathogenesis is characterized by the disruption of host cell phagosome maturation, facilitating survival and replication within host macrophages [10,11,12]. These infections often manifest covertly as chronic or subacute illnesses, characterized by non-specific symptoms such as fever and cough, and are frequently misdiagnosed due to their symptom overlap with fungal, actinomycete, or tubercular infections. Often, by the time a precise diagnosis is established, significant pulmonary lesions, typically evidenced by pulmonary consolidation with multiple cavities, have already become apparent [13]. In this case, the patient initially exhibited an isolated pulmonary mass, radiologically indicative of malignancy, and a bronchoscopic granulomatous growth, both of which are considered atypical manifestations. Conventionally, the identification of *R. equi* has relied on bacterial culturing and PCR-based detection of plasmid genes or the choE gene [14,15]. However, recent advancements in the application of microbial-targeted next-generation sequencing (tNGS) to biopsy tissues from infectious lesions have demonstrated significant diagnostic potential [16]. This case exemplifies a rare instance of successfully identifying *R. equi* in bronchoalveolar lavage fluid through tNGS, highlighting the evolving landscape of diagnostic microbiology. In the treatment of immunocompromised hosts, a regimen involving a combination of two or more antibiotics, tailored based on susceptibility testing – including agents such as vancomycin, linezolid, carbapenems, fluoroquinolones, aminoglycosides, macrolides, and rifampin – is preferred to address the complex microbial environment [17]. Conversely, for immunocompetent hosts, monotherapy employing broad-spectrum macrolides or fluoroquinolones is generally sufficient. Moreover, for infections affecting the central nervous system, the selection of multiple drugs, known for their robust CNS penetration capabilities, is recommended to ensure effective therapeutic concentrations at the site of infection [18,19,20,21]. Nevertheless, this patient presented with co-infections of *Brucella* and fungi, necessitating a sequential treatment regimen. The treatment commenced with levofloxacin combined with vancomycin, followed by azithromycin with etimicin, and concluded with minocycline with moxifloxacin. This comprehensive approach resulted in a marked improvement in both symptoms and radiological findings, demonstrating the effectiveness of a carefully tailored therapy plan in managing complex infectious diseases.

Brucellosis, attributable to the Gram-negative *Brucella* bacterium, exhibits widespread prevalence across diverse regions including the Middle East, the Mediterranean, Central Asia, and Latin America. Transmission of the disease to humans occurs primarily through direct contact with infected animals or via consumption of contaminated animal products, particularly unpasteurized milk and cheeses [22]. As the disease progresses, clinical manifestations of brucellosis vary, initially presenting with symptoms such as fever, muscular pain, arthralgia, and fatigue. In more severe cases, the infection may affect visceral organs such as the liver, spleen, and CNS, with the spine predominantly involved, leading to brucellar spondylitis [23]. This condition is characterized by typical clinical presentations, including persistent, intense lower back pain, fever accompanied by profuse sweating, and evident signs of infection in the spinal intervertebral discs and vertebral bodies [24]. Regarding treatment, it generally entails a prolonged regimen of combined antibiotics to prevent relapse [25]. From a diagnostic perspective, radiologically, it is crucial to differentiate vertebral lesions caused by brucellosis from those due to spinal tuberculosis and pyogenic spondylitis. Patients diagnosed with brucellar spondylitis typically exhibit low signal intensity on T1-weighted imaging and high signal intensity on T2-weighted imaging [26], aligning with the MRI findings observed in this case. According to the World Health Organization, a regimen of doxycycline with rifampin or doxycycline with streptomycin is recommended for treating *Brucella*, supplemented by ceftriaxone or levofloxacin in refractory or severe cases, alongside hepatoprotective and supportive therapies. In instances where conservative treatments fail, surgical intervention may be warranted for cases exhibiting spinal cord or nerve root compression, vertebral instability, or a risk of paralysis.

Co-infections involving *R. equi* and *Brucella* in immunocompetent individuals represent an extremely uncommon phenomenon, with scant reports documented in the literature. Predominantly, the majority of reported cases affect immunocompromised populations. Given this atypical presentation, further exploration into the immunological dynamics between these pathogens and the host’s immune system is necessitated, potentially offering novel insights into pathogen behavior and host resistance mechanisms. The diagnosis of this co-infection presented considerable challenges, due to symptom overlap with more prevalent diseases. Consequently, the deployment of next-generation sequencing (tNGS) in this instance enabled a swift and precise diagnosis, highlighting its essential role as a diagnostic tool in complex infectious cases. This aligns with recent research findings advocating for the broader adoption of genomic diagnostics to identify atypical pathogens [16]. In response to these diagnostic insights, our therapeutic strategy comprised a regimen of antibiotics customized according to the resistance profiles of the identified pathogens, in line with prevailing guidelines yet modified to address the specifics of this case. This methodology correlates with the scholarly consensus that personalized therapies, predicated on comprehensive microbial analysis, can enhance outcomes in infections attributable to resistant or uncommon pathogens [21,25,27]. Looking forward, future research should concentrate on the integration of genomic diagnostics into standard clinical practice and investigate the immune responses in co-infected immunocompetent hosts to elucidate the underlying mechanisms facilitating such infections.

## 4 Conclusion

In this case, the treatment regimen for the patient was rendered particularly complex due to concurrent infections with *R. equi* and *Brucella*. Such co-infections are exceptionally rare in medical literature, such that definitive guidelines for the pharmacotherapy of this unique scenario are absent. This case demonstrates that, even in individuals with normal immune function, multiple infections may manifest, presenting with atypical clinical and radiological features. This necessitates a comprehensive consideration and a thorough historical investigation by physicians when diagnosing and treating complex infectious cases. Consequently, the medical team was compelled to rely on existing literature and their clinical experience to formulate a treatment plan.
